# Complications and owner satisfaction associated with limb amputation in cats: 59 cases (2007–2017)

**DOI:** 10.1186/s12917-022-03246-z

**Published:** 2022-04-22

**Authors:** John R. Wagner, Dana M. DeSandre-Robinson, George E. Moore, Catherine A. Loughin, Micha C. Simons

**Affiliations:** 1 Department of Surgery, Long Island Veterinary Specialists, 163 S Service Rd, Plainview, NY 11803 USA; 2 Department of Surgery, Affiliated Veterinary Specialists, 9905 S US Hwy 17-92, Maitland, FL 32751 USA; 3 Present address: Department of Surgery, Veterinary Specialists of the Rockies, 774 Maleta Lane, Castle Rock, CO 80108 USA; 4 Department of Veterinary Administration, College of Veterinary Medicine, Purdue University, 625 Harrison Street, West Lafayette, IN 47907 USA; 5 Department of Small Animal Soft Tissue Surgery, Veterinary Clinical Sciences, College of Veterinary Medicine, Purdue University, 625 Harrison Street, West Lafayette, IN 47907 USA; 6 Present address: Center for Innovation in Veterinary Education and Technology, Lincoln Memorial University College of Veterinary Medicine, Harrogate, TN 37752 USA

**Keywords:** Cat limb amputation, Hind limb amputation, Forelimb amputation, Owner satisfaction, Owner survey

## Abstract

**Background:**

Limb amputation may be recommended in domestic cats following a severe injury or disease. The purpose of the study was to report the signalment, the complications, recovery outcome, owner satisfaction and expectations of domestic cats following limb amputation.

**Results:**

Medical records of 3 specialty hospitals were reviewed for cats that received a single limb amputation in a 10 year period (2007–2017). These cat owners were contacted, and 59 owners completed surveys, comprising the study population. The most common reasons for limb amputation were neoplasia (54.2%, 32/59), traumatic injury (40.7%, 24/59), bone or joint infection (3.4%, 2/59), and thromboembolism (1.7%, 1/59). Thirty-four cats (57.6%) had postoperative complications. Of the fifty-nine surveys, 52.5% reported minor complications and 5.1% reported major complications. There were no differences in postoperative complication rates for thoracic versus pelvic limb amputations. All owners reported either excellent (77.9%, 46/59), good (20.3% 12/59), or fair (1.7%, 1/59) satisfaction with the procedure. Based on their previous experiences, 84.7% (50/59) of owners would elect limb amputation if medically warranted for another pet. The remaining 15.3% of owners who would not elect limb amputation again had experienced death of their pet with a median survival time of 183 days.

**Conclusion:**

Owners reported a positive satisfaction when considering complications, recovery outcome, and expectations. This study can be used by veterinarians to guide cat owners in the decision making process of limb amputation.

**Supplementary Information:**

The online version contains supplementary material available at 10.1186/s12917-022-03246-z.

## Background

Limb amputation is a common surgical procedure performed in domestic cats and dogs. The most common reasons for performing a limb amputation include surgical removal of a neoplastic lesion, traumatic injury to the limb, congenital limb deformity, peripheral neuropathy, vascular compromise, and infection [1, 2]. When recommending limb amputation, the health of the pet (i.e., presence of comorbidities), the location and type of limb lesion, the pet’s physical ability to adapt after surgery, and owner’s perception must be considered.

There are several reports that indicate high owner satisfaction in dogs [3, 4] following limb amputation; despite this, owners continue to have a negative perception regarding limb amputation [5]. Owners are reluctant to elect limb amputation even when it may be the most appropriate, least demanding, and/or most cost-effective treatment option for their pet [5, 6]. In one study, owners objected limb amputation due to concerns of the pet’s mobility and adaptation, defense abilities, and perceived suffering [7]. In addition, owners are often concerned that the procedure may affect the pet emotionally, as extrapolated from reports in human medical literature, [8] or that there will be reduced mobility after surgery for the pet [5]. A kinematic study by Galindo-Zamora et al. showed that dogs with a pelvic limb amputation adapted quickly, and that the adaptation process began during the development of the disease before the amputation was performed [5, 9]. Furthermore, this adaptation process occurred without evidence of morphologic changes in the contralateral stifle joint examined, and with a very positive evaluation from the owner [9].

The previous literature evaluating owner satisfaction after limb amputation is primarily derived from canine studies. The owner’s decision to pursue limb amputation frequently presents an emotional situation [7]. However, canine studies report that most dog owners were satisfied with their decision for limb amputation [5, 9]. In another retrospective case series, 86% of dog owners reported they would make the same decision regarding amputation again [3]. In the same study, 88% of dog owners reported that the dog had a complete or near complete recovery [3].

It is typically assumed that high owner satisfaction outcomes have also been reported in domestic cats. In the United States, there are 2 limited reports [4, 7] including sample sizes of 18 cats and 5 cats respectively, documenting the complications, recovery outcome, and owner satisfaction and expectation following thoracic or pelvic limb amputation. However, the most recent report discussing results of a large populations of domestic cats in the United Kingdom (UK) [10] included a larger sample size of 192 cats that had undergone amputation of a single limb. This study from 2010 evaluating these measures in a population of cats from the UK reported that 89% of the cats regained normal quality of life as defined by the owner [10]. It is unknown whether owner perception would differ in the United Stations (US) as there are limited studies evaluating owner satisfaction after limb amputation in cats. Information about owner satisfaction, peri-operative complications, recovery outcome, and expectations would be helpful for the clinician and owner when electing limb amputation.

Quadrupedal locomotion has been examined in dogs more than cats. The musculoskeletal systems of quadrupeds are able to modify the gait pattern in response to painful stimuli as well as amputation. A study in clinically healthy cats revealed the percentage weight distribution, peak vertical forces, and vertical impulse were higher at the forelimbs than the hind limbs which is also consistent in healthy dogs [11]. Although cats and dogs have greater forelimb weight distribution, adaptation to thoracic limb amputation will not necessarily be more difficult than pelvic limb amputation [6]. In dogs, thoracic limb amputation has been associated with greater stress on the remaining contralateral limb, compared with pelvic limb amputation. However, few studies have investigated owner-perceived recovery or post-operative complications of cats after thoracic limb amputation, compared with pelvic limb amputation.

The purpose of this study was to report the signalment of domestic cats with limb amputation, the complications, recovery outcome, and owner satisfaction and expectations. We hypothesized most owners would report a positive satisfaction when considering limb amputation complications, recovery outcome, and expectations. We also hypothesized there would be no significant difference in post-operative complications of cats after thoracic limb amputation, compared with pelvic limb amputation according to owner survey.

## Results

### Demographics

Ninety-eight cats met the inclusion criteria during the given time period that underwent thoracic limb or pelvic limb amputation. The pets that met the inclusion criteria whose owners were unable to complete the survey were not included in the study. Fifty-nine cats were included in the study.

### Medical records review

Thirty-four were castrated males (57.6%), 23 were spayed females (38.9%), one was an intact male (1.7%), and one was an intact female (1.7%). The median age of the included cats was 9.7 years (range 0.2–18.2 years). The median age for cats that had amputations due to neoplasia was 12.6 years (range 4.0–17.0 years), trauma was 3.5 years, infection was 2.0 years (range 1.0–3.0 years), and thromboembolism was 13.0 years. There was a significant difference noted in age when comparing reasons for limb amputation (*p* = 0.001). The age distribution for cats that had an amputation due to neoplasia was significantly greater than the age distribution for cats that had an amputation due to infection (*p* = 0.010) or trauma (*p <* 0.001). The most common reason for limb amputation or injury/disease type was neoplasia (54.2%, 33/59), followed by trauma (39%, 23/59), infection (3.4% 2/59), and vascular causes (1.7%, 1/59) (Table 1).

**Table 1:** Reasons for amputation of a limb in 59 cats

Reasons for Amputation		Frequency
Neoplasia	Fibrosarcoma	9
	Osteosarcoma	5
	Lymphosarcoma	3
	Plasmacytoma	3
	Soft tissue sarcoma	3
	Anaplastic Sarcoma	2
	Hemangiosarcoma	2
	Chondrosarcoma	1
	Malignant Melanoma	1
	Myofibrosarcoma	1
	Poorly Differentiated Sarcoma	1
	Spindle cell sarcoma	1
	Synovial Cell Sarcoma	1
Trauma	Irreparable Fracture	20
	Soft tissue trauma	2
	Monoplegia	1
Infection	Osteomyelitis	2
Vascular	Thromboembolism	1

There were a variety of cat breeds including 36 (61%) domestic short hair, 10 (16.9%) domestic long hair, 5 (8.5%) Siamese, 2 (3.4%) Maine Coon, 2 (3.4%) Ragdoll, 2 (3.4%) Russian Blue, and 1 (1.7%) Bengal, and 1 (1.7%) domestic medium hair. There was no difference in injury/disease type occurrence by breed (*p* = 0.153) or sex (*p* = 0.811). Median body weight of the cats was 4.62 kg (range, 0.7 to 9.55 kg). There was no difference in injury/disease type occurrence when comparing body weight (*p* = 0.075).

Twenty-six (44.1%) cats underwent thoracic limb amputation. There were 16 (27.1%) cats with right thoracic limb amputations, and 10 (16.9%) cats with left thoracic limb amputations. All thoracic limb amputations were performed by scapulothoracic disarticulation. Eighteen amputations (30.5%) were due to neoplasia, 7 (11.9%) were due to trauma, and 1 (1.7%) was due to thromboembolism. Thirty-three (55.9%) cats underwent pelvic limb amputation. There were 18 (30.5%) cats with right pelvic limb amputations, and 15 (25.4%) cats with left pelvic limb amputations. There were 28 (47.5%) coxofemoral joint disarticulations and 5 (8.5%) mid-femoral amputations. There were 2 (3.4%) right mid-femoral amputations, and 3 (5.1%) left mid-femoral amputations. Fifteen (25.4%) were due to neoplasia, 16 (27.1%) were due to trauma, and 2 (3.4%) were due to infection.

Postoperative complications were reported in 34 (57.6%) cats and were classified as minor in 31 (52.5%) cats and major in 3 (5.1%) cats. Minor complications included mild balance difficulty in 12 (20.3%) cats, mild incisional pain in 14 (23.7%) cats, mild depressed mentation in 4 (6.8%) cats, and a combination of mild incisional pain and mild balance difficulty in one (1.7%) cat. The major complications reported were severe balance difficulty in 2 (3.4%) cats, and a combination of major decrease in appetite and severe balance difficulty in one (1.7%) cat. There were 25 (42.4%) cats with no postoperative complications. All 4 cats that were reported to have depressed mentation underwent thoracic limb amputation. There was no statistical difference between the presence of postoperative complications when comparing thoracic limb amputation to pelvic limb amputations (*p* = 0.275).

### Survey

Owners of 59 of the 98 cats were available for interview (60.2%). The median time of follow-up was 5.2 years (range: 0.4–10.9 years). All owners administered analgesic medication to their cats. The majority of owners reported excellent (59.3%) and good (37.3%) pain management with the analgesic medication (Table 2). There were no owners that reported poor pain management. The majority of owners reported excellent (69.5%) and good (27.1%) comfort level when administering postoperative pain-relief medications. When comparing pain as a postoperative complication, there was no difference in the age or injury/disease type in cats that had a limb amputation (*p* = 0.077, *p* = 0.627).

**Table 2:** Survey results

Pain management with medication	Frequency	Percentage
Excellent	35	59.3%
Good	22	37.3%
Fair	2	3.40%
**Comfort level when administering pain medication**		
Excellent	41	69.5%
Good	16	27.1%
Fair	1	1.7%
Poor	1	1.7%
**Overall owner satisfaction**		
Excellent	46	78.0%
Good	12	20.3%
Fair	1	1.7%
**General attitude / behavior**		
no change in attitude	48	81.4%
improved attitude	9	15.3%
worse attitude	2	3.4%
**Recovery Expectations**		
Better than expected	28	47.5%
As expected	25	42.8%
Worse than expected	6	10.2%
**Elect procedure on another pet**		
Yes	50	84.7%
No	4	6.7%
Unsure / depends	5	8.5%
**Recommend procedure to others**		
Yes	48	81.4%
No	0	0.0%
Unsure / depends	11	18.6%

Owners were asked to rate overall satisfaction, the pet’s general attitude, and recovery expectations. They were also asked to rate recovery expectations, whether they would elect the procedure on another pet, and whether they would recommend the procedure to others. The majority of owners reported excellent (77.9%), or good (20.3%) overall satisfaction with the procedure. The majority of cat owners reported no change in attitude (81.4%) after the procedure. Approximately half (47.5%) of the owners felt the recovery was better than expected, and less than half (42.4%) of the owners had no change in expectation. When asked if owners would elect to pursue amputation on another pet when medically recommended, the majority of owners (84.7%) would elect the procedure again. The remaining 15.3% of owners, which would not elect limb amputation again, had experienced death of their pet with a median survival time of 183 days. The majority of owners (81.4%) would also recommend the procedure to others, the remainder (18.6%) of owners were unsure. Of the 11 owners that were unsure about recommending the procedure, 9 (15.3%) cats had limb amputation due to a mass, and 4 (6.8%) cats had the limb amputation due to trauma. Seven owners (63.6%, 7/11) had cats who received a thoracic limb amputation and four owners (36.4%, 4/11) had cats who received a pelvic limb amputation.

The 59 cats included in the study had a median survival time of 850 days (range 29 to 3000 days); Twenty-four cats (40.8%) were still alive at the time of follow-up and were censored on that date. Seventeen cats (28.8%; 51.5% of cats with limb neoplasia) were euthanized or died due to evidence of metastasis, with survival times ranging from 28 days to 850 days, with a median of 365 days. Six (10.2%) cats were either euthanized or died naturally, within 1 to 8 years, secondary to unknown causes according to the owner or as recorded in the medical records. Additional causes of death were related to renal failure, unrelated neoplasia, resistant urinary tract infection, heart failure, progression of feline immunodeficiency virus disease, adrenal disease, or gastric disease.

## Discussion

Based on the results of the present study, cats that underwent limb amputation tolerated the procedure well with only minor complications including mild balance difficulty, mild incisional pain, and mildly depressed mentation. Approximately 1 in 20 cats that underwent limb amputation were reported to have major complications, which were reported as severe balance difficulty, and major decrease in appetite. The results of the owner survey were consistent with previous studies of owner satisfaction following limb amputation in cats in a UK population [10]. Previous data in canine studies indicating 91–100% of owners satisfied and 86–100% of owners would elect the procedure again parallels the results of our study in cats [3–5, 7]. This study supported our hypothesis that, within this US-based population, cat owner satisfaction would be positive when complications, recovery outcome, and expectation were considered. This study also indicated that there was no statistical difference in postoperative complications detected by owners between thoracic and pelvic limb amputation in cats, which is similar to that of the results of the previous study in cats in a UK population.

The prevalence and types of postoperative complications after limb amputation in cats have not been extensively described in the literature. In our study, the most common postoperative complication was mild balance difficulty. Although cat and dog owners have reported concerns related to anticipated problems with mobility and adaptation before limb amputation [7], postoperative complication of balance difficulty has not been previously reported in cats. Adaptation to thoracic limb amputation has revealed greater changes in ground reaction forces, impulses, and contact times of the remaining limbs and location of center of gravity compared to pelvic limb amputation in dogs [6]. In dogs, those with thoracic amputations tend to have more difficulty maintaining their balance, whereas those with pelvic limb amputation tend to have more difficulty with acceleration [12, 13]. In normal cats, gait symmetry at a walk reveals peak vertical force and vertical impulse being statistically greater in the thoracic limbs than in the pelvic limbs, which is similar to that of healthy dogs [14]. Furthermore, healthy cats have a thoracic to pelvic limb asymmetry similar to the one in healthy dogs [15]. According to our study there was no difference in recovery outcome for cats after either thoracic limb or pelvic limb amputation as perceived by owner satisfaction. The value of a body condition score would be a useful semiquantitative clinical tool to further evaluate a possible correlation with reported balance difficulty. Although not evaluated in this study, kinetic and kinematic analyses would have been valuable objective data in evaluating the new locomotion pattern and adaptation in cats before and after limb amputation.

**Fig. 1:**
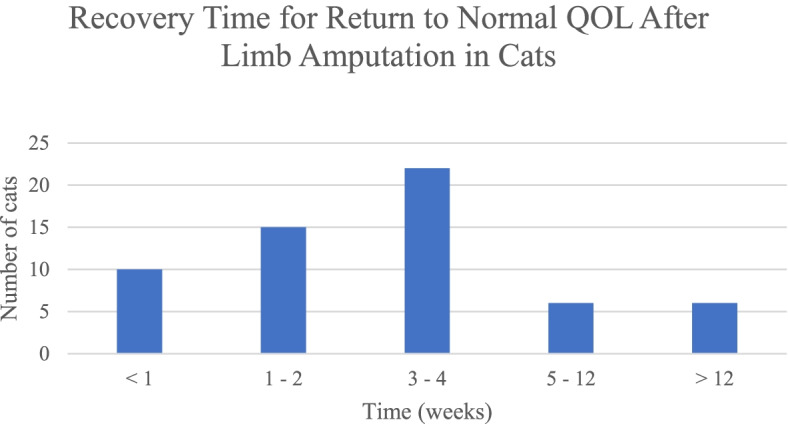
Recovery time to normal quality of life (QOL) after limb amputation in cats

The second most prevalent postoperative complication of limb amputation reported by the owner was mild pain, which has been reported in two studies [4, 10]. In our study, all cats were discharged with analgesics; however, mild pain was reported as a postoperative complication in approximately 1 in 4 cats. In a UK study in cats with limb amputation, 35% of owners observed signs of pain during recovery although 89% of all cats received analgesics after discharge [10]. In the same UK study, a significant difference existed in the time taken to return to normal quality of life where the owner perceived postoperative pain. Approximately 10% of the cats did not return to normal quality of life as defined by the owner, and approximately one third were reported to be in pain after discharge [10]. Similarly, our current study reported that 10.2% (6/59) of cats did not return to normal quality of life (Fig. 1). Although only 23.7% (14/59) cat owners observed minor signs of incisional pain, 96.6% reported either excellent (59.3%) or good (37.3%) pain management with the analgesic medication. Because there were no reported issues when administering postoperative pain-relief medications, the results may be a reflection of increased owner recognition of pain in their pet. In contrast, the reports of overall good to excellent pain management scores may reflect the evolution of analgesia protocols perioperatively. Previous historical data has indicated a lack of perioperative analgesic practices. A UK study in 1999 reported that only 74% of veterinarians dispensed analgesics beyond the immediate postoperative period in orthopedic cases [16], while perioperative use of analgesics in dogs and cats following common surgeries by Canadian veterinarians in 2001 revealed up to 12% of veterinarians did not use any analgesics [17]. Furthermore, a New Zealand study in 2005 reported only 68% of cats and 79% of dogs were dispensed additional analgesia at discharge after fracture repair [18]. More recently in 2014, attitudes regarding the use of perioperative analgesics in dogs and cats by Brazilian veterinarians revealed that cats received lower pain scores than dogs for common surgeries [19]. Recognizing pain not only requires palpation to the painful area but an appropriate understanding of the cat’s normal behavior, which should involve information from the owner [19]. In a previous canine study, 91% of owners perceived no change in their dog’s attitude after amputation,^3^ whereas in our study 6.8% of cat owners reported depressed mentation in their pet. Owner observation (i.e., depressed mentation), is an important resource when assessing and managing pain in the patient [19]. Although many studies have raised concern for perioperative pain management in cats, it is clear that the current attitudes of veterinarians regarding pain management are moving toward multimodal analgesia, which may address an owner’s concern for their pet’s pain management.

Limitations of this current study are primarily related to its retrospective design and the use of questionnaire-based surveys. These limitations included the potential for incomplete medical records and the subjectivity of surveys with having to rely on one’s memory. A prospective and standardized time to follow-up for data collection would yield a reliable degree of satisfaction. These questionnaires introduce owner bias by the level of knowledge, emotional involvement with the cat, and ability to interpret behavioral changes. However, the satisfaction of an owner represents useful information combined with the recommendations of the veterinary surgeon in the ultimate decision to treat the pet.

Previously, other postoperative complications identified in dogs have included surgical site infection, seroma, incision dehiscence, and incision revision [3, 5]. Although these additional postoperative complications were not identified in the present study in cats, it is possible owners may not accurately recall the entire postoperative period. This is a known limitation of retrospective studies of this nature. Collecting this information in a prospective study may change this information.

Although the mode of research conducted was primarily following a hypothesis-generating paradigm, the descriptive statistics have identified areas of additional research including a pet’s mobility and perceived pain. Future studies should be considered to objectively validate these findings. A prospective study including statistically significant case number and objective data, via kinetics and kinematic analyses, would be beneficial in identifying and quantifying orthopedic changes arising from amputation.

Results presented by this study suggest that limb amputation in the cat is well tolerated. The majority of owners were satisfied with the outcome post-amputation and they did not regret pursuing the procedure for their cat. However, owners should be educated on the potential for postoperative complications including balance difficulty, pain, and change in mentation, with the majority of these complications being classified as minor.

## Methods

### Case selection

Domestic cats that had undergone a limb amputation at Purdue University Veterinary Teaching Hospital (Indiana), Affiliated Veterinary Specialists (Florida), and Long Island Veterinary Specialists (New York) in a 10 year period were identified. Patient records were retrospectively reviewed and the owners of included patients were asked to complete a questionnaire. All patient records were deidentified.

### Inclusion and exclusion criteria

Domestic cats that had a single limb amputation were included in the study. Cats with concurrent orthopedic or neurologic disease affecting one or more of the remaining three limbs, or hemipelvectomy, were excluded from the study to limit variables that may affect owner satisfaction.

### Retrospective study

Data collected included patient signalment, weight, reason for amputation, the limb amputated, type of amputation performed, analgesic medication provided, postoperative complications, and histopathological results of the amputated limb in cats due to neoplasia. The time between amputation and death, when applicable, were recorded.

### Survey

After data collection, owners were contacted by telephone and interviewed using a 13-question survey to determine their satisfaction with the limb amputation procedure (Additional File 1). A second attempt was made to contact the owner if the initial contact was unsuccessful. Patients without a completed owner questionnaire were excluded from the study. The questionnaire was modified from previous veterinary investigations of quality of life [4, 5]. Data collected included owner recovery expectation, owner assessment of postoperative healing via a descriptive scale, owner satisfaction, and owner willingness to repeat and recommend the procedure. Additionally, change in the pet’s behavior and owner interaction were noted, specifically the pet’s willingness to receive human affection, recovery time to normal quality of life, and postoperative complications observed by the owner. Time to return to normal quality of life was defined by the owner. Complications were classified by severity as minor or major, and stratified into 5 recovery time groups: less than or equal to 1 week, 1 to 2 weeks, 3 to 4 weeks, 5 to 12 weeks, and greater than 12 weeks. Minor complications included mild balance difficulty (i.e., occasional difficulty entering litter box), mild pain (with tactile stimuli i.e., petting or stroking the pet, owner perceived pet had pain and/or increased sensitivity at or near the incision site), and depressed mentation. Complications were considered major if the pet displayed severe balance difficulty or pain (i.e., unable to enter litter box, unable to jump, or persistently falling over) or required prolonged hospitalization. The duration of all complications was determined by the pet’s time to return normal quality of life as defined by the owner. Information regarding the current age, survival time, and cause of death was also collected when not noted in the medical record. The institutional review determined the research survey to be exempt from IRB review, under federal human subjects research regulations 45 CFR 46.104 Category 2.

### Statistical analysis

Standard statistical methods were used to describe and analyze the numerical data. The Fisher’s exact test was used to determine associations between the injury types and breed or sex. The Kruskal-Wallis ANOVA was used to determine differences in age or weight when comparing injury type, and subsequently a Dunn test for pairwise comparison. The Wilcoxon rank sum test was used to determine a difference between age or weight when comparing presence of pain as a postoperative complication in cats after limb amputation. The Fisher’s exact test was used to assess the association between injury type and the presence of pain as a postoperative complication. The chi-square test of independence was used to compare forelimb amputation versus hindlimb amputation with postoperative complications.

Statistical analysis was performed with the aid of commercially available software. (STATA SE, v.15.1, StataCorp, College Station, TX). Values of *p* < 0.05 were considered statistically significant.

Recovery time to normal quality of life (QOL) was stratified into 5 groups: less than or equal to 1 week (16.95%, 10/59), 1 to 2 weeks (25.42%, 15/59), 3 to 4 weeks (37.29%, 22/59), 5 to 12 weeks (10.17%, 6/59), and greater than 12 weeks (10.17%, 6/59).

## Supplementary Information

**Additional file 1.** Owner Survey. This document provides the questionnaire that owners completed as part of the study.

**Additional file 2.**

## Data Availability

The datasets used and/or analyzed during the current study are available from the corresponding author.

## Abbreviations

UK: United Kingdom
US: United States
QOL: Quality of life
