# Supplementary material for: Complications and owner satisfaction associated with limb amputation in cats: 59 cases (2007–2017)
Source: BMC Vet Res. 2022 Apr 22;18:147. doi: 10.1186/s12917-022-03246-z (PMC9034555; doi:10.1186/s12917-022-03246-z)
Supplement: Supplementary file 1 — Additional file 1. Owner Survey. This document provides the questionnaire that owners completed as part of the study. [file 12917_2022_3246_MOESM1_ESM.docx]

Additional File 1:

**Hospital ________________**

**Study # _________________**

**Case No _________________**

**Owner Co # ____________________**

Owner Survey

1. Did you feel that all surgical options were explained to you?

Yes   No

If no, any explanation:

2. Did the veterinarian adequately prepare you for your pet’s procedure and recovery?

Yes   No

If no, any explanation:

 3. How do you feel the surgical site healed after the surgery?

Excellent              Good                     Fair                         Poor

If poor or fair, any explanation:

4. Did your pet appear to have pain, display abnormal behavior, or balance difficulty after the surgery?

Not at all                              Minor symptoms                         Major symptoms

What symptoms:

5. How well did the medication control your cat’s pain after the surgery?

Excellent              Good                     Fair                         Poor

If poor or fair, any explanation:

6. How comfortable were you with giving your cat pain medication after the surgery?

Excellent              Good                     Fair                         Poor

If poor or fair, any explanation:

7. How long after the surgery did your pet return to a normal quality of life?

(ie. free of clinical signs, pain relief / improved function of the pet, basic needs satisfied)

<1 week,

1 week to 2 weeks,

3 weeks to 4 weeks,

5 weeks to 12 weeks,

>12 weeks

If never, what is the residual deficit(s)?

8. What was your satisfaction with the surgery?

Excellent              Good                     Fair                         Poor

If poor or fair, any explanation:

9. Did your pet show a change in general attitude toward receiving human affection after the surgery?

Worse no change improve

If so, describe:

10. How did your pet’s recovery compare with your expectation?

As expected              better than expected mildly worse than expected worse than expected

If yes, any explanation:

11. Do you notice any change in ability after the procedure?

Yes No

If yes, is this the comment as for #8:

12. Based on your experience with your pet’s surgery, if deciding to pursue amputation on another pet, would you still elect to pursue this procedure?

Yes                         No

If no, any explanation:

13. Similarly, would you recommend other owners to pursue this procedure if medically indicated?

Yes              Maybe          No

If maybe or no, any explanation:

Additional Comments:
